# Quality of Care Provided by Family Caregivers of Older People With Dementia: Measurement and Interpretations

**DOI:** 10.1093/geroni/igaa057.496

**Published:** 2020-12-16

**Authors:** Afeez Hazzan

**Affiliations:** State University of New York at Brockport, Hilton, New York, United States

## Abstract

Family caregivers of older people living with dementia are relatives, friends, or neighbors who provide assistance related to this condition, but who are unpaid for the services they provide. Although caregiving could be personally rewarding, many caregivers report a high level of strain. Compared to caregivers of older adults who do not have dementia, family caregivers of older people living with dementia report lower quality-of-life (QoL). In a published systematic review examining the relationship between family caregiver QoL and the quality of care provided, only one study was found to be somewhat relevant. The study suggested that the primary reason for an absence of research into the link between family caregiver QoL and quality of care was the absence of a questionnaire for measuring quality of care in dementia. Therefore, any attempt to investigate the impact of caregiver QoL on the care provided to older people with dementia must first address the lack of an instrument to measure quality of care. To address this issue, we interviewed approximately 20 family caregivers in order to elicit feedback on measurements and interpretation of the quality of care provided by family caregivers of older people living with dementia. Content analysis of the interview transcripts revealed that the quality of relationships with family, caregiver availability to provide or supervise care, and availability of paid or volunteer help are important for the quality of care provided. These results have important implications, particularly for the development of an instrument to measure quality of care in dementia.

